# Warning from Canada: Latin America, South Africa and India may face an opioid epidemic in the coming years

**DOI:** 10.7189/jogh.10.010324

**Published:** 2020-06

**Authors:** Andrea D Furlan, Alexandra M Harvey, Rashmi Chadha

**Affiliations:** 1 Institute for Work & Health, Toronto ON, Canada; 2 Department of Medicine, University of Toronto, Toronto, Ontario, Canada; 3 Toronto Rehabilitation Institute, University Health Network, Toronto, Ontario, Canada,; 4 Munk School of Global Affairs, University of Toronto, Toronto, Ontario, Canada; 5 Vancouver Coastal Health, Vancouver, British Columbia, Canada; 6 Department of Family Medicine, University of British Columbia, Vancouver, British Columbia, Canada

## THE OPIOID CRISIS IN NORTH AMERICA: CAUSES AND IMPACTS

Opioid prescriptions have risen exponentially in the United States (US) and Canada over the past two decades. Between 1999 and 2011, prescriptions of oxycodone in the US increased by almost 500% [1] and those of hydrocodone nearly doubled. The overall opioid prescribing rate peaked and leveled off between 2010-2012 and has been declining since 2012. However, the amount of opioids (in morphine milligram equivalents) prescribed per person is still approximately three times higher than it was in 1999 [2]. In Canada, prescription opioid analgesics (POAs) increased in all provinces from 2005 to 2011 [3]. More than 90% of licit morphine is used by roughly 20% of the global society, primarily in the US, Canada, Germany and Australia, and approximately 66% of the world’s population have almost no access to opioids, despite having a high disease burden from conditions and illnesses known to cause pain [4]. Increased access to POAs in the US and Canada has done more than treat pain—it has spurred a regional epidemic of opioid-related deaths. It is worth pointing out that this public health emergency has complex origins and outcomes [5]; for example, the current torrent of illicit fentanyl-related deaths is primarily a product of illicit fentanyl. But, for some who are currently using illicit fentanyl, the opioid journey started with POAs to treat pain, and ultimately transitioned to illicit narcotics.

One of the driving factors that fueled liberal POA prescribing for pain in the 1990s was inflation of the benefits of opioids for pain, coupled with the narrative that POAs did not lead to harm and should be prescribed more often, including for chronic non-cancer pain [6]. Aggressive pharmaceutical advertising campaigns used strategies to sell opioids that mirrored those that were once used by the tobacco industry [7]: involvement of specialists and leaders from the field of pain medicine, overemphasis by patients’ advocacy groups on pharmacotherapy for chronic non-cancer pain, loose regulatory oversight, and the treatment of health care as a consumer good. This marketing strategy led to the ubiquitous misconception that POAs were safe and should be used to treat all chronic pain issues [6]. Terms such as “opiophobia”^7^ and pseudoaddiction^8^ were coined to discredit the growing concern surrounding POAs. Pharmaceutical manufacturers took advantage of physicians’ ethical obligations of beneficence to influence them to prescribe opioids to a larger group of chronic non-cancer pain patients, such as those suffering from low-back pain or fibromyalgia. The advertising strategy was made all the more compelling by the development of long-acting POAs with the promises of a more stable dose, even at higher doses, and the marketing strategy of “Pain as the fifth vital sign” [8]. Ultimately, the above mentioned strategies led to one of the most successful advertising campaigns in the North American pharmaceutical industry, and a major corporation recently lost trials in court for “false, misleading, and dangerous marketing campaigns” [9,10].

**Figure:**
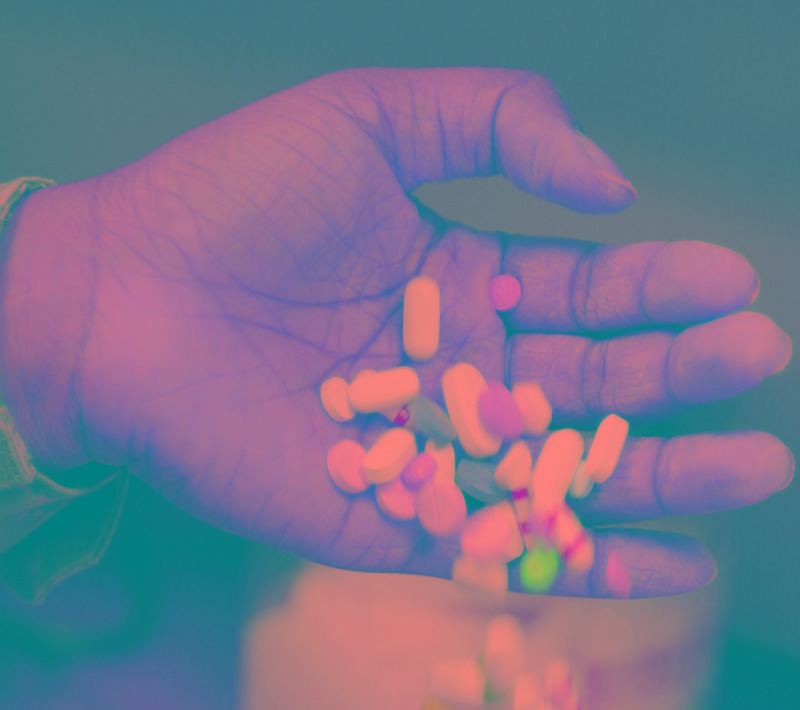
Photo: U.S. Air Force photo/Airman 1st Class Adam Grant, U.S. Air Force. Source: https://www.af.mil/News/Article-Display/Article/118588/prescription-meds-proceed-with-caution/.

## OPIOIDS GO GLOBAL: AVOIDING AN EPIDEMIC

In recent years, there has been an improved understanding in North America of the potential for harm as a result of POA consumption. Multiple good and fair-quality observational studies have been published across North America that highlight the long-term risks of opioid use and addiction [11]. Numerous guidelines for physicians prescribing opioids to patients with chronic non-cancer pain have been published in the countries that have been affected by the POA crisis [11-15] and the use of “Pain as the fifth vital sign” has largely been abandoned in the US [16]. As opioid sales have fallen in the US and Canada in response to these guidelines [17-20], but also due to the public health crisis, some of the affected pharmaceutical companies have sought out other markets for their products. Through subsidiary companies with less high-profile names (for example, in the case of Purdue Pharma, the subsidiary company is Mundipharma), pharmaceutical companies have expanded marketing of opioids for chronic pain to countries such as India, Chile, Ecuador, China, Uruguay, South Africa, Peru, and Venezuela [21].

In a July 2017 commentary, Dr Humphreys argued that the world must learn from the North American experience, and that countries must employ precautionary measures to ensure that the globalization of POAs is not closely followed by an addiction and overdose epidemic [22]. He suggested that a necessary component of precautionary efforts “should be creating clear blue water between opioid manufacturers and the formulation of prescription practice guidelines.” The “clear blue waters”—the necessary separation between opioid manufacturers and those that create prescription practice guidelines—are not currently being observed in many low- and middle-income countries.

## GUIDELINE REVIEW

A search in PubMed using the following terms (guideline or guidelines) AND (opioid or opioids) AND (Africa or Latin or India) revealed recently published guidelines/recommendations in three continents: Africa [23], Latin America [24] and South Asia [25]. A review of these documents highlighted that as Dr Humphreys feared, the “clear blue waters” are being muddied by the same tactics that were used 20 years ago in developed economies. These documents fail to cite the current epidemic in the US and Canada, nor do they reference recent guidelines on opioids for chronic pain created in North America; furthermore, the documents contain statistics that under-play the risks of opioid use disorder. Even more concerning, all three documents were developed with either direct funding from a pharmaceutical company or have lead authors who have received industry funding. **Table 1** summarizes the main points of these two recently published guidelines on opioid prescriptions in South Africa and Latin America, and a guidance document published in India.

**Table 1:** Characteristics of the guidelines and guidance document regarding opioids for chronic pain

	South Africa [23]	Latin-America [24]	India [25]
**Date**	2014	2017	2017
**Type of document**	Guideline	Guideline	Opinion paper (there are no guidelines in India)
**Title**	South African guideline for the use of chronic opioid therapy for chronic non-cancer pain	Latin-American guidelines for opioid use in chronic nononcologic pain	Addressing the barriers related with opioid therapy for management of chronic pain in India
**Goal of the document**	To provide brief and practical guidelines for the use of chronic opioid therapy (COT) in patients with CNCP. The target audience is all clinicians in primary and specialty settings who provide care for adults suffering from CNCP.	To update existing recommendations to current Latin-American reality	This review aims to identify barriers to opioid therapy for chronic pain and provides recommendations to overcome them
**Funding to develop the guideline**	The development of this guideline was supported by an unrestricted grant from Mundipharma who did not participate in the development or writing of the guideline.	Grünenthal Services, Inc. provided logistic support for the experts to be gathered. Editorial assistance was provided by Content Ed Net (Madrid, Spain) and supported by Grünenthal Services, Inc.	This study was funded by Johnson & Johnson Pvt. Ltd, India. PD (SIRO Clinpharm Pvt. Ltd) provided writing assistance and SP, PhD, CMPP (SIRO Clinpharm Pvt. Ltd) provided additional editorial support for the development of this manuscript.
**Author (or expert) affiliation with the industry**	MR has received honoraria for consultancies and non-restricted research grants from Mundipharma, Pfizer, Janssen Pharmaceutica, AstraZeneca, MSD, Eli Lilly, Aspen and Abbott Laboratories. Drs. JC and SE have received honoraria from Mundipharma. Prof. HM has received honoraria for consultancies and non-restricted research grants from Janssen Pharmaceutica, Eli Lilly, MSD and Mundipharma. Dr BS has received honoraria for consultancies and non-restricted research grants from MSD, AstraZeneca, Pfizer and Mundipharma. Dr DW has received professional fees for services to Abbott Laboratories, Adcock Ingram, Alcon Laboratories, AstraZeneca, Eli Lilly, Janssen Pharmaceutica, Mundipharma, Novartis, and Reckitt Beckiser Pharmaceuticals.	The authors have no other relevant affiliations or financial involvement with any organization or entity with a financial interest in or financial conflict with the subject matter or materials discussed in the manuscript apart from those disclosed.	PN and JAl are employees and/or shareholders of Janssen, India.
			The authors have no other relevant affiliations or financial involvement with any organization or entity with a financial interest in or financial conflict with the subject matter or materials discussed in the manuscript apart from those disclosed.

## PUBLIC HEALTH IMPLICATIONS

Guidelines for opioids for chronic pain should be developed using a comprehensive literature review, strict and careful consideration of studies, transparent methods for grading of the evidence, and unbiased expert input, among other things [26]. One crucial element of an effective guideline is a comfortable distance between those with a financial stake in the prescription of the drug and those charged with crafting its prescription guidelines. The conflicts of interest that arise when this separation is not clear are usually subtle; for example, when experts that contribute to the development of prescription guidelines are also voting members in panels that approve the guidelines’ recommendations. In other cases, the conflict of interest is overt, such as when the guideline is explicitly developed or sponsored by the opioid manufacturers themselves. When this separation is not observed, there is a strong possibility for bias in the guidelines’ recommendation in favour of the drugs being prescribed.

It is concerning that POA guidelines—documents that are crucial to the advancement of clinical practice and patient safety—are not being developed independently from the companies that profit from the sales of these medications in regions such as Latin America and South Africa; and where there are still no guidelines, the key opinion leaders are sponsored by these companies, such as in India. The three documents summarized here are intended for the management of chronic pain, which is a prevalent problem in these societies. However, these same countries have very little access to POAs for the management of cancer-related pain, palliative care and acute pain management. Therefore, high-quality pain management guidelines for opioid use in the acute care and end-of-life areas should be given first priority, as they have shown to have an important role in these settings. It is important to ensure that countries are prepared and have adequate guidelines in place to ensure the safety of their patients and prevent a crisis like the one ongoing in North America.

## References

[1] Jones C. Trends in the distribution of selected opioids by state, US, 1999–2011. Presented at National Meeting. Safe States Alliance, June 6, Baltimore, MD. 2013.

[2] Centers for Disease Control and Prevention. Prescribing data. Available: https://www.cdc.gov/DataStatistics/. Accessed: 25 January 2020.

[3] Fischer B Jones W Vojtila L Kurdyak P Patterns, changes, and trends in prescription opioid dispensing in Canada, 2005-2016. Pain Physician. 2018;21:219-28. 10.36076/ppj.2018.3.219 29871366

[4] Kunnumpurath S Julien N Kodumudi G Kunnumpurath A Kodumudi V Vadivelu N Global supply and demand of opioids for pain management. Curr Pain Headache Rep. 2018;22:34. 10.1007/s11916-018-0689-1 29619568

[5] Belzak L Halverson J The opioid crisis in Canada: a national perspective. Health Promot Chronic Dis Prev Can. 2018;38:224-33. 10.24095/hpcdp.38.6.02 29911818 PMC6034966

[6] Hadland SE Rivera-Aguirre A Marshall BDL Cerda M Association of pharmaceutical industry marketing of opioid products with mortality from opioid-related overdoses. JAMA Netw Open. 2019;2:e186007. 10.1001/jamanetworkopen.2018.6007 30657529 PMC6484875

[7] Lee S Ling PM Glantz SA The vector of the tobacco epidemic: tobacco industry practices in low and middle-income countries. Cancer Causes Control. 2012;23 Suppl 1:117-29. 10.1007/s10552-012-9914-0 22370696 PMC3332051

[8] McCaffery M Pasero CL Pain ratings: the fifth vital sign. Am J Nurs. 1997;97:15-6. 10.1097/00000446-199702000-00010 9025664

[9] Hoffman J. Johnson & Johnson ordered to pay $572 million in landmark opioid trial. New York Times. 2019 August 26, 2019.

[10] Hoffman J. Purdue Pharma tentatively settles thousands of opioid cases. New York Times. 2019 September 11, 2019.

[11] Chou R Turner JA Devine EB Hansen RN Sullivan SD Blazina I The effectiveness and risks of long-term opioid therapy for chronic pain: a systematic review for a National Institutes of Health Pathways to Prevention Workshop. Ann Intern Med. 2015;162:276-86. 10.7326/M14-2559 25581257

[12] Busse JW Craigie S Juurlink DN Buckley DN Wang L Couban RJ Guideline for opioid therapy and chronic noncancer pain. CMAJ. 2017;189:E659-66. 10.1503/cmaj.170363 28483845 PMC5422149

[13] Chou R Fanciullo GJ Fine PG Miaskowski C Passik SD Portenoy RK Opioids for chronic noncancer pain: prediction and identification of aberrant drug-related behaviors: a review of the evidence for an American Pain Society and American Academy of Pain Medicine clinical practice guideline. J Pain. 2009;10:131-46. 10.1016/j.jpain.2008.10.009 19187890

[14] Dowell D Haegerich TM Chou R cdc guideline for prescribing opioids for chronic pain–United States, 2016. JAMA. 2016;315:1624-45. 10.1001/jama.2016.1464 26977696 PMC6390846

[15] Furlan AD Reardon R Weppler C National Opioid Use Guideline G. Opioids for chronic noncancer pain: a new Canadian practice guideline. CMAJ. 2010;182:923-30. 10.1503/cmaj.100187 20439443 PMC2882451

[16] Levy N Sturgess J Mills P “Pain as the fifth vital sign” and dependence on the “numerical pain scale” is being abandoned in the US: Why? Br J Anaesth. 2018;120:435-8. 10.1016/j.bja.2017.11.098 29452798

[17] Crabtree A Rose C Chong M Smolina K Effects of the new prescribing standards in British Columbia on consumption of opioids and benzodiazepines and z drugs. Can Fam Physician. 2019;65:e231-7. 31088889 PMC6516682

[18] Morrow RL Bassett K Wright JM Carney G Dormuth CR Influence of opioid prescribing standards on drug use among patients with long-term opioid use: a longitudinal cohort study. CMAJ Open. 2019;7:E484-91. 10.9778/cmajo.20190003 31345786 PMC6658212

[19] Shields LBE Johnson TA Murphy JP Lorenz DJ Bell A Wilson KC Decline in primary care providers’ prescribing of Schedule II opioids following the implementation of federal and state guidelines. J Opioid Manag. 2019;15:111-8. 10.5055/jom.2019.0492 31343712

[20] Weiner SG Baker O Poon SJ Rodgers AF Garner C Nelson LS The effect of opioid prescribing guidelines on prescriptions by emergency physicians in Ohio. Ann Emerg Med. 2017;70:799-808e1. 10.1016/j.annemergmed.2017.03.057 28549620

[21] Ryan H, Girion L, Glover S. OxyContin goes global — “We’re only just getting started”. LA Times. 2016.

[22] Humphreys K Avoiding globalisation of the prescription opioid epidemic. Lancet. 2017;390:437-9. 10.1016/S0140-6736(17)31918-9 28792397

[23] Raff M Crosier J Eppel S Meyer H Sarembock B Webb D South African guideline for the use of chronic opioid therapy for chronic non-cancer pain. S Afr Med J. 2013;104:78-89. 10.7196/SAMJ.7316 24388094

[24] Lara-Solares A Aguayo Zamora C Amescua Garcia C Garcia JBS Berenguel Cook MDR Bonilla Sierra P Latin-American guidelines for opioid use in chronic nononcologic pain. Pain Manag. 2017;7:207-15. 10.2217/pmt-2016-0065 28166710

[25] Dureja GP Jain PN Joshi M Saxena A Das G Ahdal J Addressing the barriers related with opioid therapy for management of chronic pain in India. Pain Manag. 2017;7:311-30. 10.2217/pmt-2016-0064 28699380

[26] Brouwers MC Kho ME Browman GP Burgers JS Cluzeau F Feder G AGREE II: advancing guideline development, reporting and evaluation in health care. CMAJ. 2010;182:E839-42. 10.1503/cmaj.090449 20603348 PMC3001530

